# SREBP2 as a Novel Biomarker for Posttransplant Hepatocellular Carcinoma Recurrence: A New Prognostic Nomogram

**DOI:** 10.1155/bmri/4309464

**Published:** 2026-04-20

**Authors:** Jiali Qiu, Lei Cao, Sensheng Bi, Pengyu Han, Zhenglu Wang

**Affiliations:** ^1^ Biological Sample Resource Sharing Center, Tianjin First Central Hospital, Tianjin, China, tj-fch.com; ^2^ Tianjin Key Laboratory of Organ Transplantation, Tianjin First Central Hospital, Tianjin, China, tj-fch.com; ^3^ Tianjin Key Laboratory of Molecular Diagnosis and Treatment of Liver Cancer, Tianjin First Central Hospital, Tianjin, China, tj-fch.com

**Keywords:** hepatocellular carcinoma, liver transplantation, nomogram, recurrence prediction, SREBP2

## Abstract

Hepatocellular carcinoma (HCC) recurrence after liver transplantation (LT) remains a major cause of mortality, with current selection criteria such as the Milan and UCSF standards relying solely on tumor morphology and failing to fully account for biological aggressiveness. Emerging evidence implicates dysregulated lipid metabolism, particularly sterol regulatory element‐binding protein 2 (SREBP2), in HCC progression, yet its prognostic role in post‐LT recurrence remains unexplored. To address this gap, this study developed and validated a novel SREBP2‐integrated nomogram for improved risk stratification in 206 HCC patients undergoing LT (2015–2022), randomly split into development (*n* = 144) and validation (*n* = 62) cohorts. SREBP2 levels were quantified via ELISA (sensitivity: 0.1 ng/mL) and then incorporated into a multivariate Cox model alongside tumor number and AFP to predict recurrence‐free survival (RFS). The nomogram demonstrated superior discrimination, with C‐indices of 0.778 (development) and 0.796 (validation). High SREBP2 levels (≥ 40 ng/mL) independently predicted recurrence (HR = 1.757, *p* = 0.011), whereas decision curve analysis confirmed greater clinical net benefit across 1‐/3‐/5‐year RFS predictions. As the first study to integrate SREBP2 into LT candidate selection, this biologically informed nomogram significantly enhances recurrence prediction, offering a refined tool for transplant eligibility assessment and adjuvant therapy guidance in HCC management. By combining molecular biomarkers with clinical parameters, this approach addresses a critical unmet need in optimizing post‐LT outcomes.

## 1. Introduction

Hepatocellular carcinoma (HCC) is one of the most prevalent and lethal malignancies worldwide, representing a significant global health burden [1]. As the most common type of liver cancer, HCC ranks as the third leading cause of cancer‐related deaths globally, with its high mortality rate attributed to late diagnosis, limited treatment options, and frequent recurrence [2]. The aggressive nature of HCC highlights the urgent need for effective therapeutic strategies and improved prognostic tools to enhance patient outcomes.

Liver transplantation (LT) is the primary curative option for HCC; however, its long‐term efficacy is frequently limited by high postoperative recurrence rates, a major determinant of reduced patient survival [3]. To address this challenge, various selection criteria have been established to identify suitable candidates for LT, aiming to optimize outcomes [4]. Widely recognized standards include the Milan criteria, the University of California San Francisco (UCSF) criteria [5], and the up‐to‐seven criteria [6], each offering distinct guidelines for patient selection. Despite their clinical utility, these criteria primarily rely on tumor size and number, overlooking the potential role of molecular biomarkers in refining prognostic accuracy. Notably, serum alpha‐fetoprotein (AFP), a well‐established diagnostic and prognostic biomarker for HCC, has been incorporated into advanced selection models such as the Metroticket 2.0 criteria, demonstrating improved predictive value when combined with tumor morphological features [7]. However, AFP alone exhibits limited specificity, particularly in distinguishing HCC from benign liver conditions or other malignancies, underscoring the need for complementary biomarkers [8]. Emerging evidence underscores the critical role of dysregulated lipid metabolism in HCC progression [9], particularly through sterol regulatory element‐binding protein 2 (SREBP2), a master regulator of cholesterol biosynthesis and lipid homeostasis [10]. Given its pivotal role in tumorigenesis and metastasis [11], SREBP2 represents a promising biomarker for predicting HCC behavior and treatment outcomes.

In this study, we propose a nomogram by incorporating SREBP2 as a biomarker to enhance prognostic precision. We developed a novel nomogram model based on this extended selection criterion to predict recurrence‐free survival (RFS) following LT. By integrating clinical parameters with molecular insights, our model is aimed at providing a more comprehensive tool for risk stratification and treatment planning, ultimately improving the management of HCC patients.

## 2. Materials and Methods

### 2.1. Patients

This study included 206 HCC patients (76 with recurrence and 130 without recurrence) who underwent LT at Tianjin First Central Hospital in China from January 2015 to September 2022. All transplanted livers were procured from donation after cardiac death (DCD) donors through the Organ Procurement Organization (OPO) and allocated via the China Organ Transplant Response System (COTRS). All patients were followed‐up for at least 1 year or until recurrence. The final follow‐up date was December 31, 2023. The median follow‐up time for the entire cohort was 36.5 months (range: 12–84 months). The inclusion criteria were (1) age ≥ 18 years old; (2) postoperative histopathological examination confirms HCC; and (3) complete clinical data. The exclusion criteria included (1) combined organ transplantation; (2) history of other malignant tumors; (3) multiple LTs; (4) early postoperative mortality (≤ 30 days) caused by severe complications; and (5) missing clinical data. The assignment of development and validation groups was bases on a random sampling method implemented in Microsoft Excel to ensure unbiased allocation. First, a new column labeled “Random Number” was added to the dataset, and the formula “ = RAND( )” was applied to each row to generate a unique random number between 0 and 1. The dataset was then sorted in ascending order based on the “Random Number” column to randomize the order of the rows. Following this, the first 70% of the randomized rows were assigned to the development group, whereas the remaining 30% were allocated to the validation group. This study was conducted in accordance with the principles outlined in the 2013 revision of the Declaration of Helsinki and was approved by the Ethics Committee of Tianjin First Central Hospital (Ethics Approval Number: STEC‐TFCH‐2023‐HM‐2). Written informed consent was obtained from all participants.

### 2.2. Diagnostic Criteria for Tumor Recurrence

1. Pathological biopsy confirms that the lesion is malignant;
2. PET‐CT shows high metabolism of the lesion, which is considered malignant;
3. Laboratory tests combined with CT/MRI imaging examinations, and the determination is made after evaluation by experienced surgeons and radiologists.

The diagnosis of HCC recurrence can be established when either Criterion 1 is met or both Criteria 2 and 3 are simultaneously satisfied.

### 2.3. Laboratory Test and Pathological Characteristics

Serum AFP levels were collected from medical records. SREBP2 concentrations were detected using the Enzyme‐Linked Immunosorbent Assay (ELISA, Shanghai Enzyme‐linked Biotechnology Co. Ltd. Cat#:ml060723), with a sensitivity of 0.1 ng/mL and specificity of > 99%; the intra‐assay coefficient of variation (CV) was less than 10%, and the interassay CV was less than 15%. All assays were performed in duplicate and internal controls were included to ensure assay reproducibility.

Pathological indicators include the following: TNM stage, tumor diameter, total tumor diameter, tumor number; all surgical samples underwent standard histopathological analysis. Immediately following excision, within a 30‐min window, the specimens were preserved and sectioned along their greatest dimension. A systematic seven‐point sampling protocol was then implemented at the baseline level.

### 2.4. Statistical Analysis

Statistical analyses were performed using appropriate methods based on data characteristics. Parametric continuous variables were evaluated using Student′s *t*‐test, whereas nonparametric data were analyzed with the Mann–Whitney *U* test. Categorical variables were compared using either Fisher′s exact test or chi‐square test, depending on sample size. Diagnostic accuracy was assessed through ROC curve analysis, with numerical integration used to determine sensitivity and specificity. Optimal cutoff values for continuous variables were identified using ROC analysis combined with optimal scale regression. A significance threshold of *p* < 0.05 was applied for all statistical tests. Initial analyses were conducted using SPSS 26.0 (IBM Corporation, 2018, United States). For predictive modeling, Cox regression analysis was implemented to identify significant prognostic factors for liver cancer. All potential predictors were included to develop a simplified preoperative prediction model. The final model was presented as a nomogram, whose discriminative ability was evaluated using Harrell′s concordance index (C‐index), with values exceeding 0.75 indicating satisfactory performance. Kaplan–Meier (KM) survival curves were generated to assess time‐to‐event outcomes, stratified by significant prognostic factors, and differences between groups were compared using the log‐rank test (*p* < 0.05). To further validate the robustness of the predictive model, internal validation was performed using Bootstrap resampling with 1000 iterations. This method provides a more reliable estimate of model performance by accounting for potential overfitting and variability in the dataset. Model calibration was assessed through calibration curves, whereas clinical utility was determined using decision curve analysis (DCA). This method quantifies net benefit by comparing true positive rates against false positive rates at various probability thresholds, providing a balanced assessment of clinical outcomes. All advanced statistical analyses were performed using R software Version 4.4.2, utilizing multiple packages including “Hmisc,” “rms,” “pROC,” “rmda,” “caret,” “survival,” “survminer,” “nricens,” “boot”, “survIDIN”, and “ggDCA” for comprehensive statistical modeling and validation.

## 3. Results

### 3.1. Patient Characteristics

The baseline characteristics of the 206 enrolled patients are summarized in Table 1. The cohort was randomly divided into a development group (*n* = 144) and a validation group (*n* = 62). Comparative analysis revealed no statistically significant differences in baseline parameters between the two groups. Regarding clinical outcomes, the recurrence rates were 36.9% in the development group and 32.3% in the validation group, respectively.

**Table 1:** Patient characteristics.

Variable	Development (*n* = 144)	Validation (*n* = 62)	*p*
Recurrence			0.366
Yes	56 (38.9%)	20 (32.3%)	
No	88 (61.1%)	42 (67.7%)	
Sex			0.159
Male	125 (86.8%)	58 (93.5%)	
Female	19 (13.2%)	4 (6.5%)	
Age (years)	53.9 ± 8.4	52.4 ± 9.0	0.264
BMI	24.9 ± 4.0	24.9 ± 3.3	0.975
Waiting time for LT (months)	32.9 ± 25.9	38.3 ± 22.8	0.195
Blood type			0.133
A	36 (25%)	25 (40.3%)	
B	51 (35.4%)	16 (25.8%)	
AB	14 (9.7%)	7 (11.3%)	
O	43 (29.9%)	14 (22.6%)	
Maximum tumor diameter (cm)	4.5 ± 3.8	4.1 ± 3.7	0.421
Tumor number			0.193
1–3	89 (66.9%)	45 (76.3%)	
≥ 4	44 (33.1%)	14 (23.7%)	
Milan‐in			0.372
Yes	60 (41.7%)	30 (48.4%)	
No	84 (58.3%)	32 (51.6%)	
AFP (*μ*g/L)	3254.9 ± 9812.8	1793.5 ± 9250.4	0.345
SREBP2 (ng/mL)	46.1 ± 21.1	46.6 ± 23.3	0.878
RFS (month)	22.5 ± 17.5	25.4 ± 18.1	0.285
OS (month)	27.1 ± 15.8	30.3 ± 15.6	0.179

### 3.2. Factor Selection

Preoperative data were utilized to extract variables for univariate analysis, the results of which are summarized in Table 2. The analysis identified four factors significantly associated with recurrence:maximum tumor diameter, tumor number, AFP levels, and SREBP2 concentrations. These factors were subsequently categorized into distinct grades based on optimal scale regression analysis for further evaluation. The optimal cutoff values used for categorization were as follows: SREBP2: < 40, 40–80, and > 80 ng/mL; AFP: < 100, 100–1000, and > 1000 ng/mL; tumor number: 1–3 vs. > 3; Maximum tumor diameter: ≤ 3, 3–6, and > 6 cm.

**Table 2:** Univariate analysis results.

Variable	Recurrence	Nonrecurrence	*p*
Sex			0.416
Male	47 (83.9%)	78 (88.6%)	
Female	9 (16.1%)	10 (11.4%)	
Age (years)	53.5 ± 7.8	54.1 ± 8.8	0.674
BMI	24.9 ± 5.1	24.9 ± 3.1	0.974
Migration latency	34.7 ± 26.7	31.8 ± 25.5	0.554
Blood type			0.794
A	15	21	
B	17	34	
AB	6	8	
O	18	25	
Maximum tumor diameter (cm)	6.0 ± 5.1	3.5 ± 2.3	0.001 ^∗∗^
Maximum tumor diameter grade			0.003 ^∗∗^
≤ 3 cm	21	47	
3–6 cm	14	25	
> 6 cm	18	8	
Tumor number			< 0.001 ^∗∗∗^
1–3	26	63	
≥ 4	27	17	
Milan‐in			0.001 ^∗∗^
Yes	14	46	
No	42	42	
AFP (*μ*g/L)	6683.7 ± 14255.4	969.0 ± 3664.8	0.006 ^∗∗^
AFP grade			0.002 ^∗∗^
< 100	20	51	
100–1000	11	14	
> 1000	23	16	
SREBP2 (ng/mL)	50.8 ± 19.7	43.1 ± 21.6	0.003 ^∗∗^
SREBP2 grade			0.003 ^∗∗^
< 40	17	50	
40–80	34	33	
> 80	5	5	

^*^p < 0.05.

^**^p < 0.01.

^***^p < 0.001.

### 3.3. Constructing Recurrence Preoperative Prediction Nomogram

**Figure 1:**
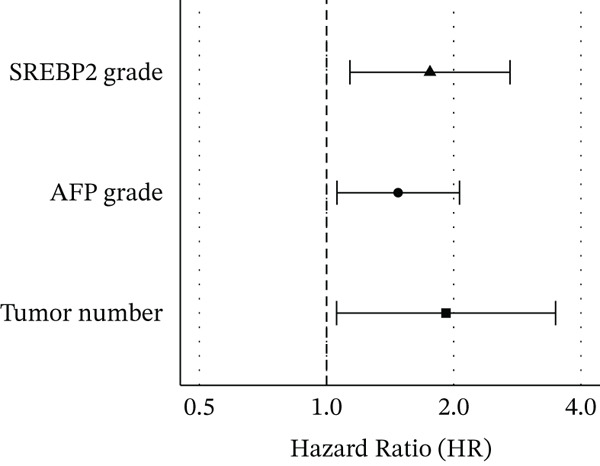
Forest plot of multivariable Cox regression results.

**Figure 2:**
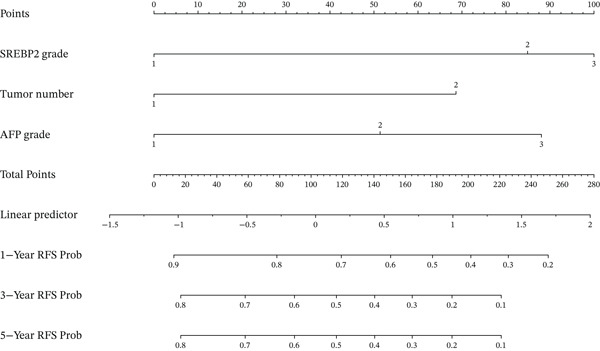
Prognostic nomogram for predicting RFS in HCC patients following LT. RFS, recurrence‐free survival; HCC, hepatocellular carcinoma; LT, liver transplantation. AFP, alpha‐fetoprotein; SREBP2, sterol regulatory element‐binding protein 2.

Four factors identified as potential risk factors of HCC recurrence through univariate analysis were subsequently incorporated into a multivariate Cox proportional hazards regression model using forward stepwise selection. Three factors (tumor number, AFP grade, and SREBP2 grade) were ultimately retained. The results of the multivariate Cox regression analysis are presented in Table 3 and illustrated in Figure 1.The analysis demonstrated that these three factors were independent risk factors for postoperative recurrence of hepatocellular carcinoma (all p<0.05), whereas maximum tumor diameter did not retain statistical significance and was excluded from the final model. We further constructed a nomogram based on these three independent risk factors (Figure 2).

**Table 3:** Multivariable Cox regression analysis results.

Variables	*β* coefficient	HR	95% CI	*p*
Tumor number	0.652	1.918	1.056–3.484	0.032 ^∗^
AFP grade	0.390	1.477	1.058–2.063	0.022 ^∗^
SREBP2 grade	0.564	1.757	1.136–2.718	0.011 ^∗^

^*^ *p* < 0.05

**Figure 4:**
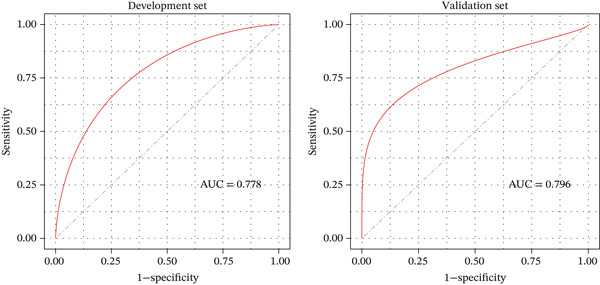
The ROC curve and AUC of the development set and the validation set. ROC, receiver operating characteristic; AUC, area under the curve.

Calibration curves demonstrated strong concordance between predicted and observed outcomes in both groups (Figure 3). The AUC values were 0.778 and 0.796 in the development and validation groups, respectively. The results indicated an acceptable discrimination capability. To further assess the robustness of the model, Bootstrap resampling with 1000 iterations was performed. The mean C‐index was 0.703 (standard deviation: 0.049), with a 95% confidence interval ranging from 0.613 to 0.807, indicating stable and reliable model performance. The ROC curve in development and validation groups was shown in Figure 4.

Figure 3(a–f) Calibration curve of 1‐, 3‐, and 5‐year RFS in the development and validation sets. RFS, recurrence‐free survival.

**Figure:**
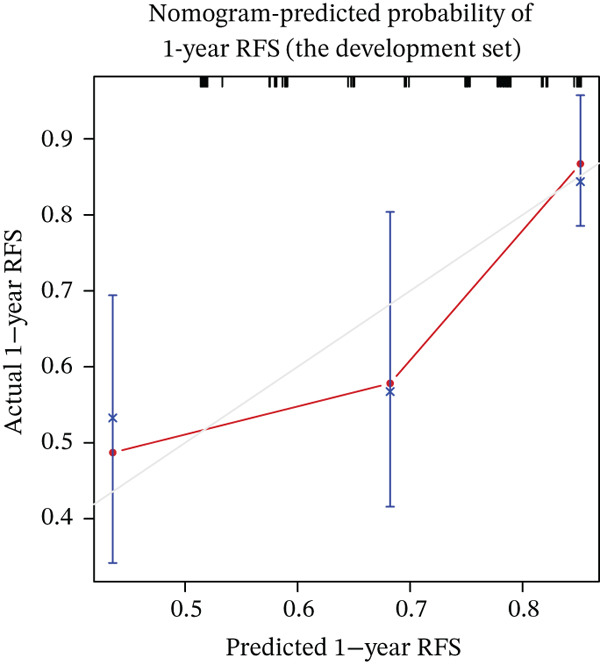
(a) Nomogram‐predicted probability of 1‐year RFS (the development set)

**Figure:**
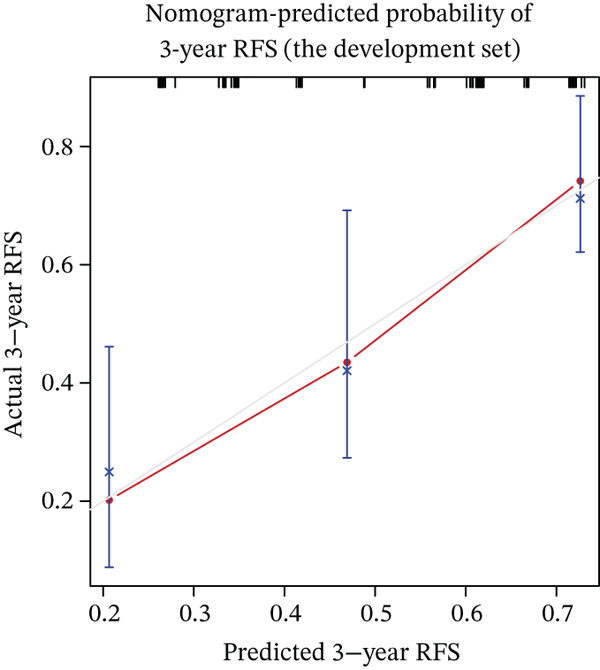
(b) Nomogram‐predicted probability of 3‐year RFS (the development set)

**Figure:**
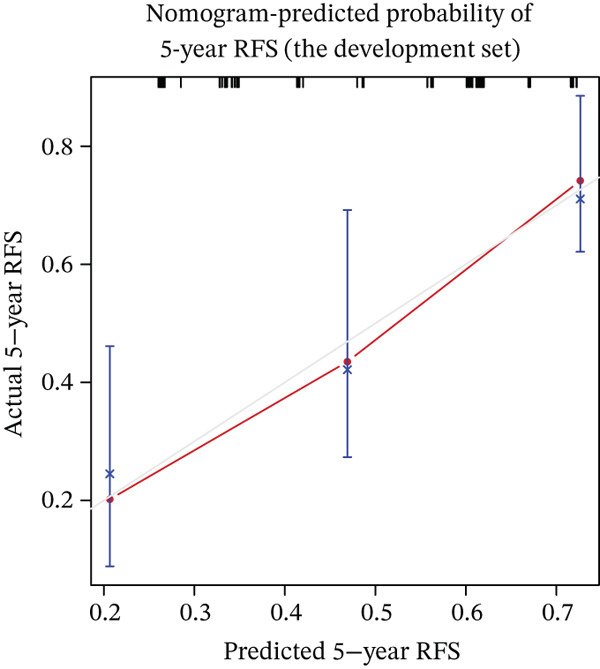
(c) Nomogram‐predicted probability of 5‐year RFS (the development set)

**Figure:**
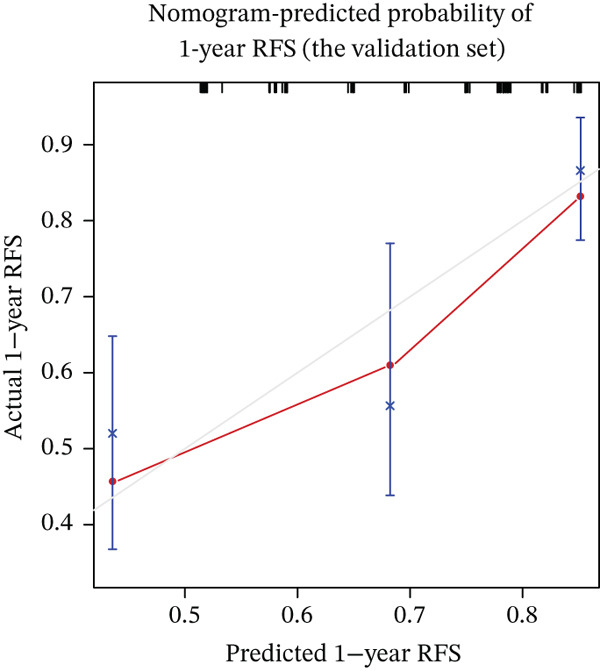
(d) Nomogram‐predicted probability of 1‐year RFS (the validation set)

**Figure:**
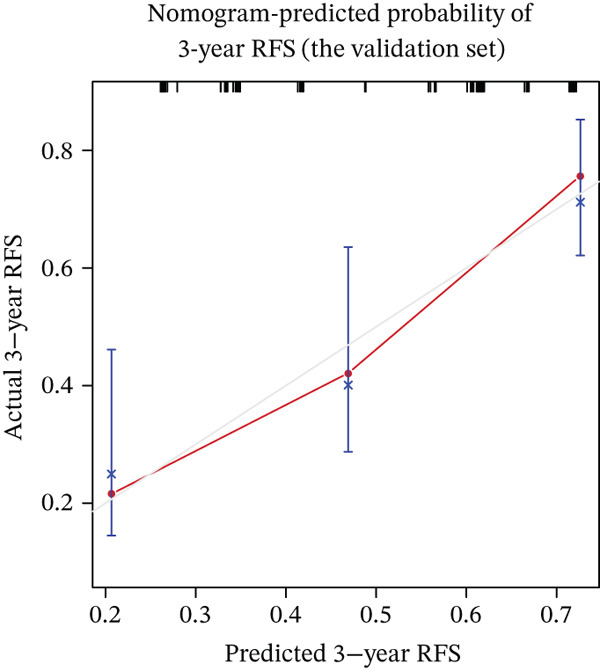
(e) Nomogram‐predicted probability of 3‐year RFS (the validation set)

**Figure:**
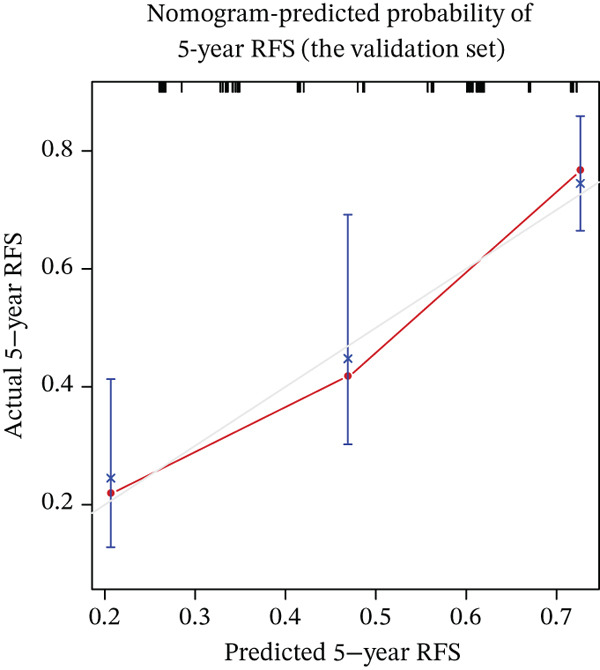
(f) Nomogram‐predicted probability of 5‐year RFS (the validation set)

**Figure 5:**
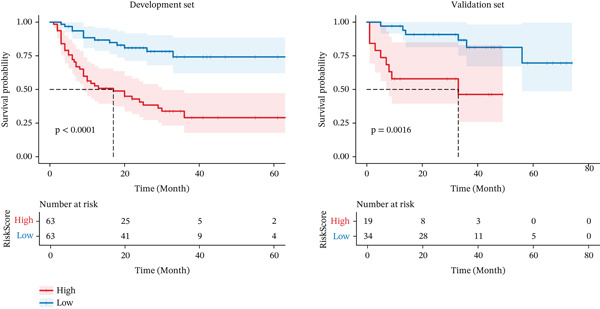
Recurrence‐free survival (RFS) analysis curves of the low‐risk and high‐risk groups in the development set and the validation set.

The nomogram assigned a continuous point score to each patient, who was subsequently stratified into low‐risk and high‐risk groups based on the median value of these scores. KM curve analysis demonstrated a statistically significant difference in RFS between the two groups (*p* < 0.01, Figure 5).

The reliability and advantages of the nomogram model were evaluated using the net reclassification index (NRI) and integrated discrimination improvement (IDI) in comparison with the traditional Milan criteria. Compared with the Milan criteria, in the development group, the NRIs for 1‐, 3‐, and 5‐year follow‐ups were 0.197 (95% CI: −0.189 to 0.485), 0.231 (95% CI: −0.364 to 0.809), and 0.231 (95% CI: −0.371 to 0.772), respectively. The corresponding IDIs were 0.043, 0.007, and 0.082. Similarly, when compared with the UCSF criteria, the nomogram showed NRIs of 0.171 (95% CI: −0.413 to 0.423), 0.028 (95% CI: −0.164 to 0.263), and 0.028 (95% CI: −0.190 to 0.251) for 1‐, 3‐, and 5‐year follow‐ups, respectively, with IDIs of 0.005, 0.073, and 0.071.

In the validation group, the NRIs for the nomogram versus the Milan criteria were 0.487 (95% CI: 0.011–0.856), 0.502 (95% CI: −0.300 to 1.027), and 0.541 (95% CI: −0.358 to 1.146) for the same timepoints, with IDIs of 0.220, 0.158, and 0.288. Against the UCSF criteria, the validation cohort demonstrated NRIs of 0.333 (95% CI: 0.000–0.778), 0.002 (95% CI: −0.289 to 0.730), and 0.002 (95% CI: −0.318 to 0.730), alongside IDIs of 0.227, 0.153, and 0.230.

**Figure 6:**
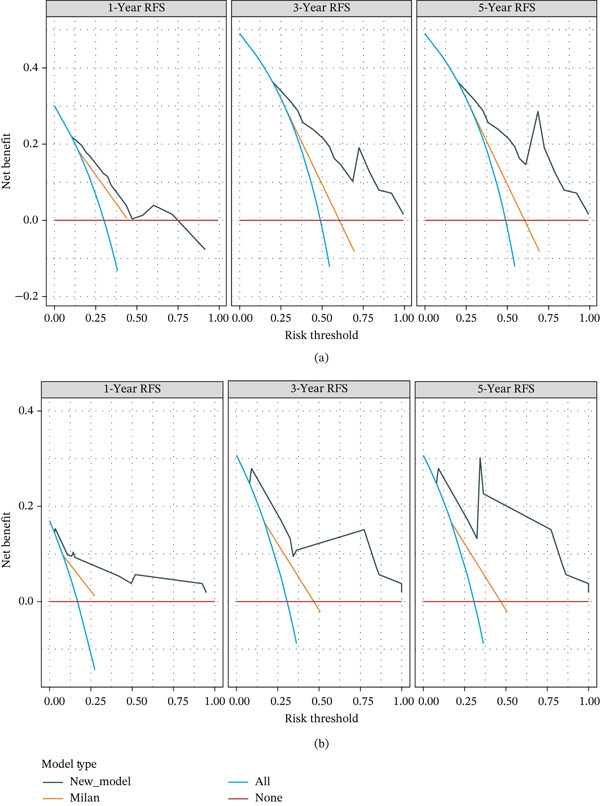
(a) DCA curves of the development set compared with the Milan criteria for 1‐, 3‐, and 5‐year RFS. (b) DCA curves of the validation set compared with the Milan criteria for 1‐, 3‐, and 5‐year RFS. DCA, decision curve analysis; RFS, recurrence‐free survival.

**Figure 7:**
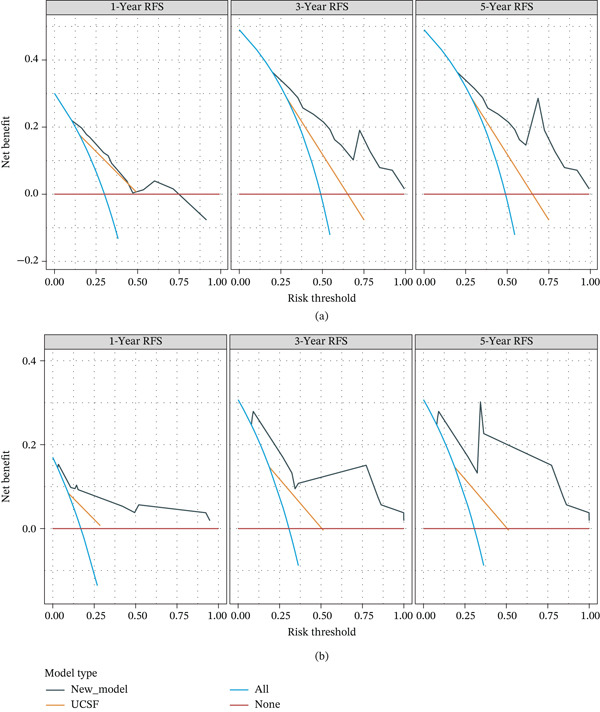
(a) DCA curves of the development set compared with the UCSF criteria for 1‐, 3‐, and 5‐year RFS. (b) DCA curves of the validation set compared with the UCSF criteria for 1‐, 3‐, and 5‐year RFS. DCA, decision curve analysis; RFS, recurrence‐free survival.

DCA was performed to compare the clinical utility of the nomogram with both the Milan and UCSF criteria. The proposed nomogram demonstrated superior net benefits for 1‐, 3‐, and 5‐year RFS compared with the Milan criteria (Figure 6) and UCSF criteria (Figure 7), particularly in intermediate‐to‐high probability thresholds, supporting its use for individualized risk stratification.

## 4. Discussion

HCC remains a significant global health burden [12], with high recurrence rates following LT severely impacting long‐term patient survival. The Milan criteria and the UCSF criteria are internationally recognized standards for LT in HCC patients. However, these criteria are often criticized for being overly stringent, potentially excluding a substantial number of patients who could benefit from transplantation [13]. Relying solely on tumor size and number to predict recurrence and metastasis has proven insufficient, as these factors do not fully capture the biological aggressiveness of HCC [14, 15]. In recent years, there has been growing interest in incorporating serum biomarkers, such as AFP, into predictive models for HCC recurrence [8, 16]. These biomarkers are readily accessible and, when combined with traditional criteria, offer improved predictive accuracy for tumor recurrence [7, 17, 18]. For instance, elevated AFP levels have been associated with higher recurrence rates and poorer outcomes, highlighting the potential of integrating serum biomarkers into existing prognostic models [19].

Emerging evidence suggests that dysregulated lipid metabolism plays a critical role in the progression of HCC [20–22]. Lipid metabolism abnormalities are increasingly recognized as key drivers of tumor growth, invasion, and metastasis [23]. Despite this, current prognostic models and selection criteria for HCC, including the Milan and UCSF standards, do not incorporate lipid metabolism‐related biomarkers. SREBP2, a master regulator of lipid metabolism, has been implicated in promoting HCC invasiveness and aggressiveness [24, 25]. SREBP2 not only regulates cholesterol metabolism but has also been shown to modulate fatty acid metabolism [26] and directly upregulate EMT transcription factors such as Twist1/Snail, promoting HCC cell migration [27]. These multifaceted oncogenic functions make SREBP2 a promising candidate biomarker for inclusion in predictive models. In this study, we expanded the Milan criteria by incorporating serum SREBP2 levels as a novel biomarker. By integrating SREBP2 into a nomogram model, we aimed to improve the prediction of RFS following LT. This approach not only refines the selection criteria for LT but also provides a more comprehensive assessment of tumor biology, potentially identifying high‐risk patients who may benefit from adjuvant therapies or closer surveillance. Our findings underscore the importance of incorporating lipid metabolism‐related biomarkers—SREBP2—into prognostic models, offering a more nuanced understanding of HCC recurrence and improving patient outcomes.

In this study, we developed a postoperative nomogram for predicting RFS by integrating two traditional clinical variables with two preoperative serum biomarkers, AFP and SREBP2. The nomogram demonstrated robust predictive performance, with C‐index values of 0.778 and 0.796 in the training and validation cohorts, respectively. The calibration curves indicated excellent agreement between the predicted and observed outcomes, suggesting that the nomogram reliably estimates the risk of recurrence. Furthermore, KM survival analysis revealed strong discriminatory ability, with clear separation of survival curves across different risk strata. To further validate the clinical utility of our nomogram, we compared it with the traditional Milan criteria and the UCSF criteria using NRI and IDI. Our nomogram outperformed both the Milan and UCSF criteria in risk prediction and classification accuracy, demonstrating its ability to accurately predict 1‐, 3‐, and 5‐year recurrence rates in HCC patients following LT. These findings highlight the potential of incorporating serum biomarkers, such as AFP and SREBP2, into prognostic models to refine risk stratification and improve clinical decision‐making.

Existing predictive models for posttransplant HCC recurrence largely rely on traditional clinical variables or emerging yet costly biomarkers. For instance, although the model by Gu et al. holds value, it incorporates a relatively large number of variables [28]. The modified AFP model for HCC with multiple nodules remains entirely dependent on clinical‐morphological indicators such as AFP and tumor size (C‐statistic 0.606) [29]. Meanwhile, models proposed by Morita [30] and Liese et al. [31] depend on the detection of complex miRNA signatures. In contrast, the nomogram developed in this study innovatively integrates SREBP2, a key regulator of lipid metabolism. This integration not only endows the model with superior discriminative performance (AUCs of 0.778 and 0.796 in the training and validation sets, respectively, outperforming the compared models) but more importantly, mechanistically unveils tumor biological aggressiveness associated with lipid metabolism—a dimension uncapturable by conventional models. Thus, our model offers a dual advantage: on a scientific level, it provides novel biological insights, enabling more precise identification of high‐risk patients and suggesting potential therapeutic targets; on a clinical‐translational level, it combines a streamlined variable set with conventional, easily accessible indicators while maintaining high predictive accuracy, thereby significantly enhancing its feasibility and generalizability for routine clinical practice.

Our nomogram demonstrates immediate clinical applicability. It can be straightforwardly integrated into the liver‐transplant candidate workup: by measuring pretransplant SREBP2 and AFP levels together with tumor number, clinicians can generate an individualized risk score to inform final selection, especially in borderline cases. We therefore propose the routine measurement of SREBP2 in LT candidates, a strategy that should be incorporated into and validated by prospective protocols, to better capture tumor biological aggressiveness. Patients classified as high‐risk by the model warrant intensified posttransplant surveillance (e.g., more frequent imaging) and consideration of adjuvant therapies, including mTOR inhibitors (which have shown promise in mitigating posttransplant recurrence) [32] or enrolment in relevant clinical trials. Notably, our findings align with emerging evidence implicating SREBP2‐mediated cholesterol biosynthesis in driving tyrosine‐kinase inhibitor (TKI) resistance [33]. A mechanistically informed strategy during the waiting period could therefore involve combining TKIs with cholesterol‐synthesis inhibitors (e.g., statins); however, this approach requires preclinical and early‐phase testing before clinical adoption. The entire risk‐adapted management framework must be validated in prospective studies.

Despite its promising results, this study has several limitations that warrant consideration. First, the retrospective nature of the study introduces inherent biases and limitations associated with the use of existing data. Retrospective analyses are susceptible to confounding factors and unmeasured variables that may influence outcomes. Second, all grafts were obtained from DCD donors, which may introduce selection bias and limit the generalizability of our findings to populations receiving grafts from other donor types. Third, the standardization of SREBP2 measurement across different laboratories requires further investigation; establishing universal assay protocols and reference ranges is crucial for multicenter validation and clinical implementation. Fourth, the relatively limited sample size may have impacted the statistical power and precision of our findings. A larger, multicenter cohort would provide more robust validation of the nomogram and enhance its generalizability. Fifth, while our model is constructed using pretransplant variables, its pretransplant application faces practical challenges, including the dynamic nature of serum biomarkers and the potential impact of downstaging therapies on tumor biology before LT. Finally, while predictive models, such as the nomogram developed in this study, offer valuable insights and tools for clinical decision‐making, they cannot replace the strong evidence derived from prospective randomized controlled trials (RCTs). RCTs remain the gold standard for establishing causal relationships and validating the efficacy of prognostic models. Nevertheless, predictive models serve as important adjuncts in clinical practice, helping clinicians identify high‐risk patients who may benefit from closer surveillance or adjuvant therapies. Future multicenter prospective studies are warranted to validate this nomogram in ethnically and geographically diverse cohorts and explore the integration of additional biomarkers or imaging modalities to further enhance its predictive accuracy.

## 5. Conclusion

This study presents a novel approach to predicting HCC recurrence after LT by integrating SREBP2, a key regulator of lipid metabolism, with traditional clinical parameters. Our findings demonstrate the importance of incorporating molecular biomarkers into prognostic models to better reflect tumor biology and improve clinical decision‐making. The developed nomogram offers a practical tool for risk stratification and personalized treatment planning, addressing current limitations in posttransplant management. This work represents a significant step toward precision medicine in HCC care, highlighting the potential of combining biological insights with clinical parameters to optimize patient outcomes.

## Author Contributions

Jiali Qiu conceived and designed the experiments, performed the experiments, analyzed the data, prepared figures and/or tables, and approved the final draft. Lei Cao authored or reviewed drafts of the paper and approved the final draft. Sensheng Bi and Pengyu Han performed the experiments and approved the final draft. Zhenglu Wang conceived and designed the experiments, authored or reviewed drafts of the paper, and approved the final draft.

## Funding

This study was supported by Tianjin Natural Science Foundation (22JCYBJC01230) and the Tianjin Key Medical Discipline (Specialty) Construction Project (TJYXZDXK‐3‐026C and TJYXZDXK‐3‐006A).

## Disclosure

All authors approved the manuscript. The authors have reviewed and edited the output and take full responsibility for the content of this publication.

## Ethics Statement

The studies involving human participants were reviewed and approved by the ethics committees of Tianjin First Central Hospital (Approval No. STEC‐TFCH‐2023‐HM‐2). Written informed consent was obtained from all individual participants included in the study prior to their participation.

## Conflicts of Interest

The authors declare no conflicts of interest.

## Data Availability

The data that support the findings of this study are available on request from the corresponding author. The data are not publicly available due to privacy or ethical restrictions.

## References

[1] Singal A. G. , Kanwal F. , and Llovet J. M. , Global Trends in Hepatocellular Carcinoma Epidemiology: Implications for Screening, Prevention and Therapy, Nature Reviews Clinical Oncology. (2023) 20, no. 12, 864–884, 10.1038/s41571-023-00825-3. 37884736

[2] European Association For The Study Of The Liver , Clinical Practice Guidelines on the Management of Hepatocellular Carcinoma, Journal of Hepatology. (2025) 82, no. 2, 315–374, 10.1016/j.jhep.2024.08.028. 39690085

[3] Tabrizian P. , Holzner M. L. , Mehta N. , Halazun K. , Agopian V. G. , Yao F. , Busuttil R. W. , Roberts J. , Emond J. C. , Samstein B. , Brown R. S. , Najjar M. , Chapman W. C. , Doyle M. M. , Florman S. S. , Schwartz M. E. , and Llovet J. M. , Ten-Year Outcomes of Liver Transplant and Downstaging for Hepatocellular Carcinoma, Journal of the American Medical Association Surgery. (2022) 157, no. 9, 779–788, 10.1001/jamasurg.2022.2800. 35857294 PMC9301590

[4] Sha M. , Wang J. , Cao J. , Zou Z. H. , Qu X. Y. , Xi Z. F. , Shen C. , Tong Y. , Zhang J. J. , Jeong S. , and Xia Q. , Criteria and Prognostic Models for Patients With Hepatocellular Carcinoma Undergoing Liver Transplantation, Clinical and Molecular Hepatology. (2025) 31, no. Supplement 2024, S285–S300, 10.3350/cmh.2024.0323. 39159949 PMC11925443

[5] Yao F. Y. , Ferrell L. , Bass N. M. , Watson J. J. , Bacchetti P. , Venook A. , Ascher N. L. , and Roberts J. P. , Liver Transplantation for Hepatocellular Carcinoma: Expansion of the Tumor Size Limits Does not Adversely Impact Survival, Hepatology. (2001) 33, no. 6, 1394–1403, 10.1053/jhep.2001.24563, 2-s2.0-0034991518. 11391528

[6] Mazzaferro V. , Llovet J. M. , Miceli R. , Bhoori S. , Schiavo M. , Mariani L. , Camerini T. , Roayaie S. , Schwartz M. E. , Grazi G. L. , Adam R. , Neuhaus P. , Salizzoni M. , Bruix J. , Forner A. , De Carlis L. , Cillo U. , Burroughs A. K. , Troisi R. , Rossi M. , Gerunda G. E. , Lerut J. , Belghiti J. , Boin I. , Gugenheim J. , Rochling F. , Van Hoek B. , Majno P. , and Metroticket Investigator Study Group , Predicting Survival After Liver Transplantation in Patients With Hepatocellular Carcinoma Beyond the Milan Criteria: A Retrospective, Exploratory Analysis, Lancet Oncology. (2009) 10, no. 1, 35–43, 10.1016/S1470-2045(08)70284-5, 2-s2.0-57749191711. 19058754

[7] Mazzaferro V. , Sposito C. , Zhou J. , Pinna A. D. , De Carlis L. , Fan J. , Cescon M. , Di Sandro S. , Yi-Feng H. , Lauterio A. , Bongini M. , and Cucchetti A. , Metroticket 2.0 Model for Analysis of Competing Risks of Death After Liver Transplantation for Hepatocellular Carcinoma, Gastroenterology. (2018) 154, no. 1, 128–139, 10.1053/j.gastro.2017.09.025, 2-s2.0-85038262730. 28989060

[8] Norman J. S. , Li P. J. , Kotwani P. , Shui A. M. , Yao F. , and Mehta N. , AFP-L3 and DCP Strongly Predict Early Hepatocellular Carcinoma Recurrence After Liver Transplantation, Journal of Hepatology. (2023) 79, no. 6, 1469–1477, 10.1016/j.jhep.2023.08.020. 37683735 PMC10998694

[9] Hall Z. , Chiarugi D. , Charidemou E. , Leslie J. , Scott E. , Pellegrinet L. , Allison M. , Mocciaro G. , Anstee Q. M. , Evan G. I. , Hoare M. , Vidal-Puig A. , Oakley F. , Vacca M. , and Griffin J. L. , Lipid Remodeling in Hepatocyte Proliferation and Hepatocellular Carcinoma, Hepatology. (2021) 73, no. 3, 1028–1044, 10.1002/hep.31391. 32460431

[10] Plebanek M. P. , Xue Y. , Nguyen Y. V. , DeVito N. C. , Wang X. , Holtzhausen A. , Beasley G. M. , Theivanthiran B. , and Hanks B. A. , A Lactate-SREBP2 Signaling Axis Drives Tolerogenic Dendritic Cell Maturation and Promotes Cancer Progression, Science Immunology. (2024) 9, no. 95, eadi4191, 10.1126/sciimmunol.adi4191. 38728412 PMC11926670

[11] Che L. , Chi W. , Qiao Y. , Zhang J. , Song X. , Liu Y. , Li L. , Jia J. , Pilo M. G. , Wang J. , Cigliano A. , Ma Z. , Kuang W. , Tang Z. , Zhang Z. , Shui G. , Ribback S. , Dombrowski F. , Evert M. , Pascale R. M. , Cossu C. , Pes G. M. , Osborne T. F. , Calvisi D. F. , Chen X. , and Chen L. , Cholesterol Biosynthesis Supports the Growth of Hepatocarcinoma Lesions Depleted of Fatty Acid Synthase in Mice and Humans, Gut. (2020) 69, no. 1, 177–186, 10.1136/gutjnl-2018-317581, 2-s2.0-85064008952. 30954949 PMC6943247

[12] Huang D. Q. , Mathurin P. , Cortez-Pinto H. , and Loomba R. , Global Epidemiology of Alcohol-Associated Cirrhosis and HCC: Trends, Projections and Risk Factors, Nature Reviews Gastroenterology & Hepatology. (2023) 20, no. 1, 37–49, 10.1038/s41575-022-00688-6. 36258033 PMC9579565

[13] Costentin C. E. , Bababekov Y. J. , Zhu A. X. , and Yeh H. , Is It Time to Reconsider the Milan Criteria for Selecting Patients With Hepatocellular Carcinoma for Deceased-Donor Liver Transplantation?, Hepatology. (2019) 69, no. 3, 1324–1336, 10.1002/hep.30278, 2-s2.0-85061249433. 30229978

[14] Hoang T. P. T. , Schindler P. , Börner N. , Masthoff M. , Gerwing M. , von Beauvais P. , De Toni E. N. , Lange C. M. , Trebicka J. , Morgül H. , Seidensticker M. , Ricke J. , Pascher A. , Guba M. , Ingrisch M. , Wildgruber M. , and Öcal O. , Imaging-Derived Biomarkers Integrated With Clinical and Laboratory Values Predict Recurrence of Hepatocellular Carcinoma After Liver Transplantation, Journal of Hepatocellular Carcinoma. (2023) 10, 2277–2289, 10.2147/JHC.S431503. 38143909 PMC10740736

[15] Prasad K. R. , Young R. S. , Burra P. , Zheng S.-S. , Mazzaferro V. , Moon D. B. , and Freeman R. B. , Summary of Candidate Selection and Expanded Criteria for Liver Transplantation for Hepatocellular Carcinoma: A Review and Consensus Statement, Liver Transplantation. (2011) 17, no. supplement 2, S81–S89, 10.1002/lt.22380, 2-s2.0-80053267175. 21748847

[16] Li Q. , Wei Y. , Zhang T. , Che F. , Yao S. , Wang C. , Shi D. , Tang H. , and Song B. , Predictive Models and Early Postoperative Recurrence Evaluation for Hepatocellular Carcinoma Based on Gadoxetic Acid-Enhanced MR Imaging, Insights into Imaging. (2023) 14, no. 1, 10.1186/s13244-022-01359-5. PMC9826770 36617581

[17] Mehta N. , Heimbach J. , Harnois D. M. , Sapisochin G. , Dodge J. L. , Lee D. , Burns J. M. , Sanchez W. , Greig P. D. , Grant D. R. , Roberts J. P. , and Yao F. Y. , Validation of a Risk Estimation of Tumor Recurrence After Transplant (RETREAT) Score for Hepatocellular Carcinoma Recurrence After Liver Transplant, Journal of the American Medical Association Oncology. (2017) 3, no. 4, 493–500, 10.1001/jamaoncol.2016.5116, 2-s2.0-85018557318. PMC5395317 27838698

[18] Cucchetti A. , Serenari M. , Sposito C. , Di Sandro S. , Mosconi C. , Vicentin I. , Garanzini E. , Mazzaferro V. , De Carlis L. , Golfieri R. , Spreafico C. , Vanzulli A. , Buscemi V. , Ravaioli M. , Ercolani G. , Pinna A. D. , and Cescon M. , Including mRECIST in the Metroticket 2.0 Criteria Improves Prediction of Hepatocellular Carcinoma-Related Death After Liver Transplant, Journal of Hepatology. (2020) 73, no. 2, 342–348, 10.1016/j.jhep.2020.03.018. 32201284

[19] Dong B. , Zhang H. , Duan Y. , Yao S. , Chen Y. , and Zhang C. , Development of a Machine Learning-Based Model to Predict Prognosis of Alpha-Fetoprotein-Positive Hepatocellular Carcinoma, Journal of Translational Medicine. (2024) 22, no. 1, 10.1186/s12967-024-05203-w. PMC11092049 38741163

[20] Paul B. , Lewinska M. , and Andersen J. B. , Lipid Alterations in Chronic Liver Disease and Liver Cancer, Journal of Hepatology Reports. (2022) 4, no. 6, 100479, 10.1016/j.jhepr.2022.100479. PMC9034302 35469167

[21] Huang B. , Song B.-L. , and Xu C. , Cholesterol Metabolism in Cancer: Mechanisms and Therapeutic Opportunities, Nature Metabolism. (2020) 2, no. 2, 132–141, 10.1038/s42255-020-0174-0. 32694690

[22] Greenlee J. D. , Subramanian T. , Liu K. , and King M. R. , Rafting Down the Metastatic Cascade: The Role of Lipid Rafts in Cancer Metastasis, Cell Death, and Clinical Outcomes, Cancer Research. (2021) 81, no. 1, 5–17, 10.1158/0008-5472.CAN-20-2199. 32999001 PMC7952000

[23] Bian X. , Liu R. , Meng Y. , Xing D. , Xu D. , and Lu Z. , Lipid Metabolism and Cancer, Journal of Experimental Medicine. (2020) 218, no. 1, e20201606, 10.1084/jem.20201606. PMC7754673 33601415

[24] Wang S. , Zhou Y. , Yu R. , Ling J. , Li B. , Yang C. , Cheng Z. , Qian R. , Lin Z. , Yu C. , Zheng J. , Zheng X. , Jia Q. , Wu W. , Wu Q. , Chen M. , Yuan S. , Dong W. , Shi Y. , Jansen R. , Yang C. , Hao Y. , Yao M. , Qin W. , and Jin H. , Loss of Hepatic FTCD Promotes Lipid Accumulation and Hepatocarcinogenesis by Upregulating PPAR*γ* and SREBP2, Journal of Hepatology Reports. (2023) 5, no. 10, 100843, 10.1016/j.jhepr.2023.100843. PMC10477690 37675273

[25] Tang W. , Zhou J. , Yang W. , Feng Y. , Wu H. , Mok M. T. S. , Zhang L. , Liang Z. , Liu X. , Xiong Z. , Zeng X. , Wang J. , Lu J. , Li J. , Sun H. , Tian X. , Yeung P. C. , Hou Y. , Lee H. M. , Lam C. C. H. , Leung H. H. W. , Chan A. W. H. , To K. F. , Wong J. , Lai P. B. S. , Ng K. K. C. , Wong S. K. H. , Wong V. W. S. , Kong A. P. S. , Sung J. J. Y. , and Cheng A. S. L. , Aberrant Cholesterol Metabolic Signaling Impairs Antitumor Immunosurveillance Through Natural Killer T Cell Dysfunction in Obese Liver, Cellular & Molecular Immunology. (2022) 19, no. 7, 834–847, 10.1038/s41423-022-00872-3. 35595819 PMC9243114

[26] Triki M. , Rinaldi G. , Planque M. , Broekaert D. , Winkelkotte A. M. , Maier C. R. , Janaki Raman S. , Vandekeere A. , Van Elsen J. , Orth M. F. , Grünewald T. G. P. , Schulze A. , and Fendt S. M. , mTOR Signaling and SREBP Activity Increase FADS2 Expression and Can Activate Sapienate Biosynthesis, Cell Reports. (2020) 31, no. 12, 107806, 10.1016/j.celrep.2020.107806. 32579932 PMC7326293

[27] Zhang F. , Gao J. , Liu X. , Sun Y. , Liu L. , Hu B. , Wang Z. , Shi J. , Guo W. , and Zhang S. , LATS-Regulated Nuclear-Cytoplasmic Translocation of SREBP2 Inhibits Hepatocellular Carcinoma Cell Migration and Invasion via Epithelial-Mesenchymal Transition, Molecular Carcinogenesis. (2023) 62, no. 7, 963–974, 10.1002/mc.23538. 37042569

[28] Gu Y.-G. , Xue H.-Y. , Ma E.-S. , Jiang S.-R. , Li J.-H. , and Wang Z.-X. , A Novel Nomogram to Predict the Recurrence of Hepatocellular Carcinoma After Liver Transplantation Using Extended Selection Criteria, Hepatobiliary & Pancreatic Diseases International. (2025) 24, no. 3, 252–260, 10.1016/j.hbpd.2024.06.002. 38890106

[29] Wang J. , Bao J. , Wang R. , Hong J. , Zhang L. , Que Q. , Xu S. , Wu Y. , Zhan Q. , Liu Y. , Liu J. , Zheng S. , Ling S. , and Xu X. , The Predictive Value of the Modified AFP Model for Liver Transplantation Outcomes in Multinodular Hepatocellular Carcinoma Patients, World Journal of Surgical Oncology. (2023) 21, no. 1, 10.1186/s12957-023-02994-y. PMC10041809 36967432

[30] Morita K. , Shirabe K. , Taketomi A. , Soejima Y. , Yoshizumi T. , Uchiyama H. , Ikegami T. , Yamashita Y. , Sugimachi K. , Harimoto N. , Itoh S. , Ikeda T. , and Maehara Y. , Relevance of MicroRNA-18a and MicroRNA-199a-5p to Hepatocellular Carcinoma Recurrence After Living Donor Liver Transplantation, Liver Transplantation. (2016) 22, no. 5, 665–676, 10.1002/lt.24400, 2-s2.0-84964780492. 26783726

[31] Liese J. , Peveling-Oberhag J. , Doering C. , Schnitzbauer A. A. , Herrmann E. , Zangos S. , Hansmann M. L. , Moench C. , Welker M. W. , Zeuzem S. , Bechstein W. O. , and Ulrich F. , A Possible Role of MicroRNAs as Predictive Markers for the Recurrence of Hepatocellular Carcinoma After Liver Transplantation, Transplant International. (2016) 29, no. 3, 369–380, 10.1111/tri.12733, 2-s2.0-84958551974. 26697811

[32] Grigg S. E. , Sarri G. L. , Gow P. J. , and Yeomans N. D. , Systematic Review With Meta-Analysis: Sirolimus- or Everolimus-Based Immunosuppression Following Liver Transplantation for Hepatocellular Carcinoma, Alimentary Pharmacology & Therapeutics. (2019) 49, no. 10, 1260–1273, 10.1111/apt.15253, 2-s2.0-85064484448. 30989721

[33] Mok E. H. K. , Leung C. O. N. , Zhou L. , Lei M. M. L. , Leung H. W. , Tong M. , Wong T. L. , Lau E. Y. T. , Ng I. O. L. , Ding J. , Yun J. P. , Yu J. , Zhu H. L. , Lin C. H. , Lindholm D. , Leung K. S. , Cybulski J. D. , Baker D. M. , Ma S. , and Lee T. K. W. , Caspase-3-Induced Activation of SREBP2 Drives Drug Resistance via Promotion of Cholesterol Biosynthesis in Hepatocellular Carcinoma, Cancer Research. (2022) 82, no. 17, 3102–3115, 10.1158/0008-5472.CAN-21-2934. 35767704

